# Alzheimer's Disease, time to turn the tide

**DOI:** 10.18632/aging.101581

**Published:** 2018-10-13

**Authors:** Stefano L. Sensi

**Affiliations:** 1 Center of Excellence on Aging and Translational Medicine, University G. d’Annunzio of Chieti-Pescara, Chieti-Pescara, Italy; 2 Department of Neuroscience, Imaging, and Clinical Sciences, University G. d’Annunzio of Chieti-Pescara, Chieti-Pescara, Italy; 3 Departments of Neurology and Pharmacology, Institute for Mind Impairments and Neurological Disorders, University of California, Irvine, CA 92697, USA

**Keywords:** neuroprotection, BDNF, trophic factors, amyloid, tau, dementia, diabetes, exenatide, GLP-1

**Figure 1:**
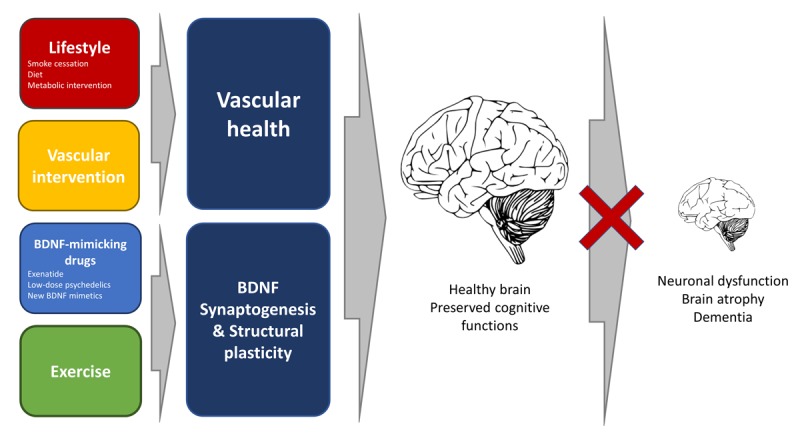
The pictogram illustrates multiple preventative actions to delay brain aging and delay the onset of dementia

Aging is the major risk factor for neurodegenerative diseases and dementia. Between 2015 and 2050, the number of older people living in higher income countries is forecast to increase by just 56% supporting the fear of an incoming dementia epidemic. Time to act is now. In Alzheimer’s disease (AD), we are gaining great ground in the field of early diagnosis, but disease-modifying drugs are still missing. While many studies have been focused on the pathogenic role of β-amyloid (Aβ) dysmetabolism, recent preclinical and clinical findings revealed a more complex picture. Some authors have called for a rejection of the amyloid hypothesis [1], new and old players like tau-related pathology microglia activation and neuroinflammation are now looming on the horizon, but the core of the issue is that the reductionist approach that has dominated modern medicine should be abandoned. We need an epistemological leap forward, a change in paradigm, and embrace a complex view of the disease state as a condition resulting from the converging failure of many health-controlling systems and networks. A condition that, for each subject, is shaped by the combination of the individual “omic” lookout and its modulation by the environment. Moreover, we need to leave behind the illusion that a single bullet/intervention can cure and adopt a *systems biology* approach [2]. AD is a multifactorial condition in which, along with Aβ accumulation, the convergence of many genetic, environmental, vascular, metabolic, and inflammatory factors promote the disease. All these conditions find fertile ground, inside and outside of the Central Nervous System (CNS), provided by the aging process. In that respect, approaches targeting co-morbidity factors are becoming promising as, at least, a third of AD cases are strongly dependent on the concerted activity of modifiable factors like low education, midlife hypertension, midlife obesity, diabetes, physical inactivity, smoking, and depression [3]. One promising area of early intervention concerns the vascular system. The timing of intervention is also critical. Systematic reviews have revealed that cardiovascular factors go out of range in young adulthood or middle age (< 65 years), but not necessarily in late life (≥ 75 years) and studies indicate that such early “early on” alterations are the ones associated with an increased AD risk. The impact of targeting these midlife individuals is high. A 10% reduction in the exposure to cardio-metabolic risk factors in midlife could prevent up to 1.1 million cases of AD per year worldwide. So far, non-pharmacological single-domain interventions have yielded suboptimal results, but combined preventative approaches have produced a significant reduction in the incidence of dementia [4]. On the metabolic front, an emerging and promising area concerns the role of insulin-signaling in dementia. Insulin is secreted by the pancreas but also by the brain where the hormone acting as a neurotrophic factor critically modulates neuronal survival, synaptic plasticity and controls the molecular pathways underlying learning and memory processes [5]. Decreased insulin sensitivity is found upon brain aging, and defective insulin signaling has been reported in subjects affected by Mild Cognitive Impairment (MCI) and AD patients. Glucagone is an endogenous insulinotropic hormone that participates in the homeostatic regulation of insulin and glucose. Like insulin, the activation of the Glucagon-like peptide-1 receptor (GLP-1R) impacts on neuronal excitability, synaptic plasticity, and memory. These effects are primarily obtained through the activation of cAMP response element-binding protein (CREB), the induction of the expression of the Brain-Derived Neurotrophic Factor (BDNF), and the activation of its tropomyosin-related kinase B receptor (TrkB). GLP-1 analogues have been tested in preclinical models of neurodegeneration and clinical trials. In particular, exenatide, a long-lasting GLP-1R agonist approved for T2DM treatment, is under evaluation in trials targeting AD and produced promising effects in Parkinson’s disease (PD; NCT01971242) [6]. In a 2013 study (Bomba et al., Cell Death Dis. 2013), we have tested a 6-month treatment with exenatide in Presenilin-1 Knock-In mice, a preclinical model of amyloid-independent neuronal dysfunction and found that the molecule promotes beneficial effects on Short- and Long-Term Memory performances. More recently (Bomba et al., Neurobiol Aging. 2018), we have also shown that a 2-month exenatide treatment results in enhanced cognitive performances in adult mice. The study has great translational value as the timeframe of intervention, mice at 10-12 month of age, matches the mid-life stage of humans, thereby opening a critical window of opportunity for preventative intervention. Exenatide exerted positive effects through to increased expression of BDNF and TrkB and the downstream activation of BDNF-related signalling. These results pinpoint the importance of targeting the neurotrophic systems and BDNF in particular. High levels of brain BDNF expression are associated with a decreased rate of cognitive decline and a milder course of AD. Mature BDNF (mBDNF) promotes neurogenesis, synatogenesis as well as neuroprotection upon adulthood and against AD-related neurodegeneration [7,8]. BNDF levels are increased by physical activity. Intriguingly, in a recent study (Ly et al. Cell Rep., 2018). These findings open a promising new frontier and indicate the need to further research into the pharmacological modulation of this neurotrophic pathway. If we take up the challenge, it is conceivable that, in the near future, along with exercise, vascular and metabolic interventions, a mix of new or rediscovered BDNF mimetic drugs will give us the therapeutic options that we have been missed for so long. It is also conceivable that more focused efforts aimed at the affordable synthesis of human BDNF or BDNF mimetics will trigger the therapeutic revolution that, in the treatment of diabetes, we have witnessed upon the introduction of human insulin (Figure 1).

Courage, curiosity, and free spirit are all we need to turn the tide finally.
